# Variability in body weight and body composition and cognitive trajectories in older adults in the United States

**DOI:** 10.1002/oby.24309

**Published:** 2025-07-03

**Authors:** Ashley C. Flores, Alexandra M. Wennberg, Cindy W. Leung, Muzi Na

**Affiliations:** ^1^ Department of Nutritional Sciences The Pennsylvania State University University Park Pennsylvania USA; ^2^ Unit of Epidemiology Institute of Environmental Medicine, Karolinska Institutet Stockholm Sweden; ^3^ Department of Nutrition, Harvard T.H. Chan School of Public Health Boston Massachusetts USA

## Abstract

**Objective:**

This study examined the associations of variability and patterns in BMI, body weight (BW), and waist circumference (WC) with cognitive decline.

**Methods:**

A total of 4304 participants (aged ≥65 years) from the National Health and Aging Trends Study between 2011 around 2021 were analyzed. Adjusted mixed‐effect models assessed BW and body composition variability metrics linked to cognitive function *z* scores over 11 years, including standard deviation (SD), coefficient of variation (CV), root mean square error, last‐to‐first assessment change groups, and overall pattern over the follow‐up.

**Results:**

Participants in the highest SD variability quartile had the fastest cognitive decline (β = −0.036 [95% CI: −0.044 to −0.028] *z* scores per year) compared with the lowest variability BMI quartile (β = −0.019 [95% CI: −0.027 to −0.010] *z* scores per year, *p* values for interaction, < 0.001). Similar trends were observed for BMI CV and root mean square error, BW SD and CV, and WC CV. Compared with the stable or gain group, participants with ≥5% loss in BMI and BW had the fastest cognitive decline (both *p* values for interaction, < 0.0002). The cognitive decline rates among the stable, loss, gain and cycling patterns in BMI, BW, or WC were not significantly different.

**Conclusions:**

Greater variability and loss in BW and body composition were linked to accelerated cognitive decline in older adults.

**Study Importance**

**What is already known?**

- Age‐related cognitive decline impacts key functions and quality of life in older adults.
- Variability in body weight (BW) and BMI has been associated with cognitive decline in older adults.
- Research on the variability and directionality of BW and body composition change, particularly in older age, is limited, with inconsistent use of metrics across studies.

**What does this study?**

- Higher variability in BMI, BW, and waist circumference (WC) over 11 years was linked to accelerated cognitive decline in older adults.
- BMI, BW, and WC loss patterns (≥5% decrease) and BW cycling (≥5% decrease and increase) were associated with the fastest cognitive decline.
- Sensitivity analyses excluding dementia, diabetes, and adjusting for grip strength confirmed robustness; no interactions were found by sex or weight loss intentionality.

**How might these results change the direction of research or the focus of clinical practice?**

- This study highlights the importance of monitoring changes in anthropometrics in older adults to address cognitive decline, suggesting that stable BW and body composition are important for promoting cognitive health.
- These findings may inform preventive strategies aimed at maintaining stable BMI, BW, and WC to mitigate cognitive decline in older adults.

## INTRODUCTION

The aging population in the United States is expected to nearly double by 2030 [1], with an estimated one in five American individuals projected to be aged 65 years or older [2]. This demographic shift has significant implications for cognitive health in older age [3]. Age‐related cognitive decline affects executive function, visuospatial skills, language, processing speed, and memory [4]. Understanding these changes is crucial for maintaining functional independence [5], activities of daily living [6], and quality of life [7]. Identifying modifiable risk factors that predict longitudinal cognitive change among older adults has become increasingly important.

Recent research has highlighted the relationship between body weight (BW), as a potential modifiable factor, and cognitive health [8, 9]. For instance, obesity during midlife is strongly linked to cognitive impairment and the development of neurodegenerative diseases later in life [10]; however, the relationship between obesity in late life and cognitive health is less well understood. Conversely, weight loss in older adults (defined as a reduction of ≥4% to 5% over several years) has been associated with brain atrophy [11] and cognitive impairment [12]. Increasing recognition of changes in anthropometric measures, such as body mass index (BMI) and waist circumference (WC), highlights their importance for cognitive function [13, 14], with central obesity, measured by WC [15], identified as a key, although less‐studied, factor in cognitive outcomes [16]. In prior research, a limited number of metrics (ranging from one to four) have been used to quantify variability and directional changes (increases or decreases) in BMI and body weight in older adults, but the approaches have not been consistent across studies [8, 14, 17]. However, a significant research gap exists in comprehensively evaluating changes in BW and body composition and applying these different variability and pattern metrics to understand their unique contributions to cognitive decline in older adults. Additionally, the intentionality of weight loss should be considered, as unintentional weight loss or unexplained weight loss may imply impending mortality and underlying conditions [18], which may have different implications for cognitive function compared with intentional weight loss.

This current study aims to bridge this gap by using a comprehensive range of metrics to assess the magnitude, pattern, and direction of annual BMI, BW, and WC changes and their impact on cognitive decline in older adulthood. Therefore, our objective is to investigate and compare the associations between multiple patterns and variability in BW and body composition measures with the trajectory of cognitive decline over 11 years in a nationally representative sample of Medicare beneficiaries aged 65 years and older in the United States.

## METHODS

### Study design and participants

Data were drawn from the 2011 to 2021 National Health and Aging Trends Study (NHATS) [19]. Initiated in 2011 (baseline), 8245 older adults enrolled in the first NHATS round and were drawn from the Medicare enrollment file using a stratified three‐stage sample design, including oversampling individuals by older ages and non‐Hispanic Black individuals [20]. Starting in 2011, annual in‐person interviews collected detailed information on participants. Due to the COVID‐19 pandemic, in‐person data collection shifted to telephone interviews for round 10 of NHATS (2020) [19]. In round 11 of NHATS (2021), interviews were conducted either by telephone or in person with performance activities involving a home visit [19]. Detailed information regarding the changes made to the interview procedures and questionnaire items have been reported elsewhere [19].

To be eligible for inclusion in our study, participants needed complete BMI, BW, and WC data for at least three rounds to calculate the variability measures and complete cognitive assessment data for at least two rounds to estimate the cognitive change. Participants with diagnosed or probable dementia reported at baseline (*n* = 511) were excluded.

### Standard protocol approvals, registrations, and patient consents

NHATS was approved by the Johns Hopkins Bloomberg School of Public Health institutional review board, and all participants provided informed consent. This study used secondary data analysis of deidentified data from the NHATS data and met the criteria for nonhuman subject research, exempting it from The Pennsylvania State University institutional review board review.

### Assessment of cognitive function

NHATS objectively assessed cognition by three domains (i.e., memory, orientation, and executive function) during in‐person or telephone (round 10 only) interviews at each survey round [19]. Memory was evaluated by immediate and delayed recall tasks with a 10‐item word list. The total memory score included the immediate and delayed recall performance tasks, ranging from 0 to 20, with higher scores indicating more words that participants recalled. The orientation domain of cognitive functioning incorporated orientation to date and the naming of the US president and vice president. A point was given for every correct answer, producing a total orientation score from 0 to 8, with a score of 8 representing greater orientation. To gauge executive function, participants were instructed to complete the Clock Drawing Test and were scored on a scale from 0, i.e., “not recognizable as a clock” to 5, i.e., “an accurate depiction of a clock.” The baseline mean (standard deviation [SD]) of memory, orientation, and executive function scores was used to derive domain‐specific *z* scores in all rounds. The combined cognitive function *z* score was derived from the mean of the three domain‐specific *z* scores for each round.

### Assessment of BW and body composition

Participants were asked to self‐report their current height in feet and inches and weight in pounds [19]. We calculated BMI in kilograms per meters squared and BW in kilograms. A flexible tape measure was placed around the participant's waist, and the objective measurement was recorded on the tape to the nearest one‐quarter inch after the participant exhaled. WC was measured at each survey round by trained research personnel and was not administered in round 10 due to COVID‐19 restrictions [19].

### Variability indicators and classification in BW and body composition

To assess the intraindividual variability of BMI, BW, and WC over time, we used three variability indices, including the following: 1) SD, 2) coefficient of variation (CV), and 3) root mean square error (RMSE). CV was computed by dividing the SD by the mean. RMSE was calculated as the deviation around the linear slope of the anthropometric measure in relationship to wave adjusting for baseline age. The three variability indices for each anthropometric exposure were then categorized into quartiles, with quartile 1 representing the lowest variability and quartile 4 representing the highest variability.

In addition, two pattern categories were categorized according to the following: 1) the difference between the last assessment to the first assessment, as commonly used in clinical trials [21] and 2) the overall pattern over time to capture the trajectory of change [22]. Specifically, the following three groups were defined for the difference between the last assessment to the first assessment: 1) stable (<5% change), 2) loss (≥5% decrease), and 3) gain (≥5% increase). For the overall pattern, we defined the following four groups based on the differences from the prior year and from the baseline: 1) stable (<5% change), 2) loss (≥5% decrease), 3) gain (≥5% increase), and 4) cycling (≥5% decrease and increase).

### Covariates

The following baseline sociodemographic and health variables were collected from NHATS 2011: age (65–69, 70–74, 75–79, 80–84, 85–89, and ≥90 years), sex (male or female), race and ethnicity (White, non‐Hispanic Black, non‐Hispanic, and other), education levels (less than high school, high school, and post‐high school education), and regular smoker status (yes or no), defined as ever smoked cigarettes regularly (at least 1 cigarette a day). Grip strength was measured twice at baseline using a Jamar Plus digital hand dynamometer and the averaged score was created according to the NHATS guideline, in which 0 was the worst score and 5 was the best score. In our study, intentional weight loss (yes or no) was ever reported in only    *n* = 2088 participants across all rounds of NHATS. A subsample analysis was conducted to explore the potential differential impact of intentionality of weight loss of the associates being examined.

Time‐varying covariates were assessed annually, except for income information which was collected biennially. These data included marital status (married, living with partner, not married, widowed, separated, or divorced) and income quintiles based on total imputed income (imputation methods have been described elsewhere [19, 23]); current smoking status (yes or no), defined as smoking cigarettes now; current depression score (computed using the validated two‐item Patient Health Questionnaire, ranged from 0 to 6); current anxiety score (derived using the validated two‐item Generalized Anxiety Disorder Scale, ranged from 0 to 6); and self‐reported diagnosed chronic conditions (yes or no for each condition), including heart disease, diabetes, hypertension, stroke, and cancer.

### Statistical analysis

After adjusting for the stratified three‐stage sampling design to analyze baseline sample characteristics, we calculated the weighted percentage and mean (SE) for categorical and continuous variables.

We used mixed‐effect models with random intercept without accounting for sampling weights to examine how the variability indicators were associated with cognitive *z* scores and time. To compare cognitive decline across the groups, we included the categorical‐by‐continuous interaction term between the nutritional variability status group and time. Our analyses were adjusted for the baseline time‐invariant and time‐varying covariates, the baseline anthropometric measure being examined, and the number of follow‐ups. In the analyses of BW and WC, we further adjusted for baseline height and BMI, respectively.

We conducted sensitivity analyses to test the robustness of our results, including the following: 1) excluding data collected during the COVID‐19 pandemic (rounds 10 and 11), 2) excluding participants who developed dementia during the follow‐up (*n* = 458), 3) excluding participants who developed diabetes during the follow‐up, and 4) additionally adjusted for baseline grip strength. We also included a three‐way interaction term of the variability or pattern variable, wave, and sex or intentional weight loss (yes or no) to test whether the cognitive decline slope differed by sex or intentionality of weight loss. All statistical analyses were conducted using Stata/SE, version 17.0 (StataCorp LLC).

## RESULTS

Our study included 4304 eligible NHATS participants (Figure S1), followed over a mean of 6.55 years, and representing a population of 21,018,794 older adults. The median (interquartile range [IQR]) number of completed annual cognitive tests was 8 (4,11), with 73.7% of participants completing 5 or more cognitive assessments. The median number (IQR) of nutritional assessments was 8 (3,11) for BMI, BW, and WC. The median time between the first and the last nutritional assessment was 7.8 years. The baseline characteristics of the study/analytic population are shown in Table 1.

**TABLE 1:** Baseline characteristics of the NHATS older adults^a^ (*N* = 4304)

	*n*	% or mean (SE)
Age groups, %	4304	
65–69 y		30.6
70–74 y		26.3
75–79 y		19.2
80–84 y		14.0
85–89 y		7.3
≥90 y		2.7
Sex, %	4304	
Male		43.3
Female		56.7
Race and ethnicity, %	4296	
White, non‐Hispanic		84.5
Black, non‐Hispanic		7.4
Hispanic		5.5
Other		2.6
Education, %	4298	
<High school diploma		17.6
High school diploma		26.5
>High school diploma		55.9
Smoking status, %	4304	
Never smoker		47.8
Ever smoker		44.5
Current smoker		7.7
Marital status, %	4300	
Married, living with partner		59.2
Not married, widowed, separated, divorced		40.8
Imputed income quintiles, %	4304	
1 ($0–$9000)		17.4
2 ($9001–$17,402)		16.6
3 ($17,403–$30,000)		19.2
4 ($30,001–$55,000)		22.4
5 (>$55,000)		24.4
Depression, score^b^	4285	2.8 (0.02)
Anxiety, score^c^	4295	2.8 (0.02)
Heart disease, %	4298	16.2
Diabetes, %	4304	22.8
Hypertension, %	4298	63.4
Stroke, %	4301	8.4
Cancer, %	4302	25.5
BMI (kg/m^2^)	4247	27.9 (0.11)
BW (lb)	4274	174.0 (0.84)
WC (in)	4140	39.4 (0.22)

Abbreviations: BW, body weight; NHATS, The National Health and Aging Trends Study; WC, waist circumference.

^a^ Adjusted for the complex sampling scheme.

^b^ Depressive symptoms score was calculated based on the validated two‐item Patient Health Questionnaire.

^c^ Anxiety score was calculated based on the validated two‐item Generalized Anxiety Disorder Scale.

Table 2 presents the estimated trajectories of cognitive function *z* scores according to the variability of metrics quartiles. Across the three different variability metrics for BMI, a consistent trend for the fastest decline in cognition was observed in the highest variability quartiles, demonstrated by the greater negative β values in Quartile 4 compared to Quartile 1. The magnitude of cognitive decline for the β values in Quartile 4 was 2.5 times (−0.037 vs. −0.015) for RMSE, 3.3 (−0.039 vs. −0.012) for SD, and 4.9 (−0.044 vs. −0.009) for CV greater than those in Quartile 1. Similar to BMI, a greater decline in cognitive function is observed with higher variability quartiles for BW and WC. For BW, the magnitude of cognitive decline in Quartile was 2 to 3 times greater than in Quartile 1, whereas for WC, the magnitude ranges from 1.5 to 2.3 times greater.

**TABLE 2:** Predicted trajectory of cognitive function *z* score between 2011 and 2021 in NHATS older adults by nutritional status variability quartiles.

		Quartile 1	Quartile 2	Quartile 3	Quartile 4	*p*‐value for interaction^d^
	*n*	β (95% CI)	β (95% CI)	β (95% CI)	β (95% CI)	
BMI^a^						
SD	4191	−0.012 (−0.018 to −0.006)	−0.019 (−0.024 to −0.014)	−0.027 (−0.033 to −0.022)	−0.039 (−0.044 to −0.033)	<0.0001
CV	4191	−0.009 (−0.015 to −0.004)	−0.020 (−0.026 to −0.015)	−0.025 (−0.030, −0.020)	−0.044 (−0.050 to −0.038)	<0.0001
RMSE	4196	−0.015 (−0.020 to −0.009)	−0.017 (−0.022 to −0.011)	−0.026 (−0.031 to −0.021)	−0.037 (−0.043 to −0.031)	<0.0001
BW^a^, ^b^						
SD	4195	−0.015 (−0.020 to −0.009)	−0.019 (−0.025 to −0.014)	−0.028 (−0.034 to −0.023)	−0.035 (−0.041 to −0.029)	<0.0001
CV	4195	−0.013 (−0.019 to −0.008)	−0.018 (−0.023 to −0.012)	−0.027 (−0.033 to −0.022)	−0.042 (−0.048 to −0.036)	<0.0001
RMSE	4199	−0.014 (−0.020 to −0.008)	−0.018 (−0.023 to −0.012)	−0.029 (−0.034 to −0.023)	−0.034 (−0.040 to −0.028)	<0.0001
WC^a^, ^c^						
SD	3920	−0.013 (−0.019 to −0.007)	−0.020 (−0.026 to −0.015)	−0.023 (−0.028 to −0.017)	−0.029 (−0.035 to −0.023)	0.0001
CV	3920	−0.014 (−0.020 to −0.008)	−0.018 (−0.024, −0.012)	−0.021 (−0.026, −0.015)	−0.032 (−0.038, −0.027)	<0.0001
RMSE	3925	−0.015 (−0.021 to −0.009)	−0.022 (−0.027 to −0.016)	−0.024 (−0.029 to −0.018)	−0.022 (−0.028 to −0.017)	0.0508

Abbreviations: BW, body weight; NHATS, National Health and Aging Trends Study; RMSE, root mean square error; WC, waist circumference.

^a^ Adjusted for baseline covariates including age, sex, race and ethnicity, education level, regular smoker status, nutritional status being examined, number of follow‐ups, and time‐varying covariates for marital status, imputed income quintiles, current smoking status, depression score, anxiety score, and current diagnosis status of heart disease, diabetes, hypertension, stroke, or cancer.

^b^ Further adjusted for baseline height.

^c^ Further adjusted for baseline BMI.

^d^ *p* value for interaction indicates the *p* value of the interaction between quartile group and time and tested at a significance level of 0.05.

The associations between anthropometric measures from the last‐to‐first assessment change groups and cognitive decline are demonstrated in Table 3. Loss of ≥5% from the last‐to‐first assessment of BMI, BW, and WC was associated with accelerated rates of cognitive decline in comparison with participants with stable anthropometric measures (<5%). Participants who lost ≥5% in the last‐to‐first BMI assessment had a doubled cognitive decline rate of −0.031 *z* scores per year (95% CI: −0.036, −0.027), as opposed to the stable participants whose estimated decline rate was −0.015 *z* scores per year (95% CI: −0.020 to −0.011; *p* values for interaction <0.0001). Compared with the stable participants whose decline rate was −0.016 *z* scores per year (95% CI: −0.021 to −0.012), loss of ≥5% in the last‐to‐first BW also had a nearly doubled cognitive decline rate of −0.028 *z* scores per year (95% CI: −0.032 to −0.023; *p* value for interaction <0.0001). To a smaller extent, participants of the WC loss group also had faster cognitive decline (β = −0.027; 95% CI: −0.035 to −0.018 *z* score per year) compared with the WC stable change group (β = −0.019; 95% CI: −0.026 to −0.012 *z* scores per year; *p* = 0.0085).

**TABLE 3:** Predicted trajectory of cognitive function *z* score between 2011 and 2021 in NHATS older adults by nutritional status last‐to‐first assessment change groups.

		Loss	Stable	Gain	*p* value for interaction^d^
	*n*	β (95% CI)	β (95% CI)	β (95% CI)	
BMI^a^	4203	−0.031 (−0.036 to −0.027)	−0.015 (−0.020 to −0.011)	−0.019 (−0.026 to −0.013)	<0.0001
BW^a^, ^b^	4105	−0.028 (−0.032 to −0.023)	−0.016 (−0.021 to −0.012)	−0.019 (−0.027 to −0.012)	<0.0001
WC^a^, ^c^	3876	−0.027 (−0.035 to −0.018)	−0.019 (−0.026 to −0.012)	−0.021 (−0.028 to −0.013)	0.0085

Abbreviations: BW, body weight; NHATS, National Health and Aging Trends Study; WC, waist circumference.

^a^ Adjusted for baseline covariates including age, sex, race and ethnicity, education level, regular smoker status, nutritional status being examined, number of follow‐ups, and time‐varying covariates for marital status, imputed income quintile, current smoker status, depression score, anxiety score, and current diagnosis status of heart disease, diabetes, hypertension, stroke, or cancer.

^b^ Further adjusted for baseline height.

^c^ Further adjusted for baseline BMI.

^d^ *p* value for interaction indicates the *p* value of the interaction between last‐to‐first assessment change group and time and tested at a significance level of 0.05.

Table 4 presents the associations between the patterns of anthropometric measures, additionally considering cycling, defined by the differences from the prior year and baseline, with cognitive decline. We observed participants with a BW cycling pattern had a cognitive decline rate nearly double that of the stable pattern group, with β values of −0.026 *z* scores per year versus −0.016 *z* scores per year (*p* value for interaction =0.0331). Participants with loss or cycling patterns in BMI or WC demonstrated higher cognitive decline rate, followed by the gain and stable groups; however, the differences among the four groups were not significant (both *p* values for interaction >0.12).

**TABLE 4:** Predicted trajectory of cognitive function *z* score between 2011 and 2021 in NHATS older adults by nutritional status pattern.

		Stable	Loss	Gain	Cycling	*p* value for interaction^d^
	*n*	β (95% CI)	β (95% CI)	β (95% CI)	β (95% CI)	
BMI^a^	4180	−0.017 (−0.026 to −0.008)	−0.024 (−0.030 to −0.018)	−0.017 (−0.024 to −0.011)	−0.024 (−0.028 to −0.019)	0.1264
BW^a^, ^b^	4189	−0.016 (−0.023 to −0.008)	−0.023 (−0.028 to −0.018)	−0.018 (−0.025 to −0.011)	−0.026 (−0.030 to −0.021)	0.0331
WC^a^, ^c^	3906	−0.011 (−0.020 to −0.001)	−0.023 (−0.031 to −0.015)	−0.020 (−0.027 to −0.014)	−0.022 (−0.026 to −0.017)	0.1268

Abbreviations: BW, body weight; NHATS, National Health and Aging Trends Study; WC, waist circumference.

^a^ Adjusted for baseline covariates including age, sex, race and ethnicity, education level, regular smoker status, nutritional status being examined, number of follow‐ups, and time‐varying covariates for marital status, imputed income quintile, current smoker status, depression score, anxiety score, and current diagnosis status of heart disease, diabetes, hypertension, stroke, or cancer.

^b^ Further adjusted for baseline height.

^c^ Further adjusted for baseline BMI.

^d^ *p* interaction indicates the *p* value of the interaction between nutritional status pattern group and time and tested at a significance level of 0.05.

Removing COVID‐19 data did not materially change our results (data not shown). After excluding participants who developed dementia (Table S1) or diabetes during follow‐up (Table S2), or additionally adjusting for grip strength (Table S3), the observed trends remained consistent, with higher quartiles of variability associated with greater cognitive decline, similar to findings in the full sample. The magnitude was slightly reduced in the subsample excluding dementia cases. Loss in the last‐to‐first assessment, based on changes in anthropometric measures, remained associated with the fastest cognitive decline rates, even after exclusions. However, the association was less pronounced in the sensitivity analysis excluding dementia cases (Table S4) and slightly more pronounced in models with additional adjustment for grip strength (Table S6). Sensitivity analyses confirmed the findings from the full sample that the cognitive decline only differed by the four patterns in BW but not BMI or WC after excluding dementia or diabetes cases (Tables S7 and S8) or after further adjustment for grip strength. We observed significant interactions for BW CV (*p* value for interaction =0.034) and WC SD (*p* value for interaction =0.0115; Table S10). By the intentionality of weight loss status, we observed significantly different cognitive decline trajectories for BMI pattern (*p* value for interaction =0.0398; Table S10). None of the other interactions examined was significant.

## DISCUSSION

Our study of older adults examined the associations between variations in BMI, BW, and WC and cognitive decline from 2011 to 2021. The results indicate that higher variability in BW and body composition over time is linked to accelerated cognitive decline. Additionally, our study suggests that experiencing a loss pattern of anthropometric measures and a cycling pattern in BW is particularly detrimental for cognitive decline. By analyzing multiple dimensions of anthropometric change, including variability and pattern, this study fills a research gap in the existing literature. Our findings emphasize the importance of maintaining a stable BW and body composition for cognitive health in older adulthood.

Our study demonstrates that greater variability in BMI, BW, and WC, despite different indices measured (i.e., SD, CV, and RMSE), was associated with faster cognitive decline, highlighting the importance of tracking BW, height, and body composition and monitoring variability in central obesity [24]. Each metric offers distinct insights into how anthropometric measures change with cognitive decline. The mean value heavily influences SD, whereas CV normalizes the variability relative to the mean and is advantageous for individuals with varying mean nutritional status values [22, 25]. RMSE, which is less affected by short‐term fluctuations, unlike CV and SD, quantifies the magnitude of deviation from a linear trend of nutritional status over time [22]. These metrics underscore the significance of a multifaceted approach by considering each index's strengths and limitations to understand anthropometric variability with cognitive decline. The consistent associations across variability metrics for the three BW and body composition measures provide evidence that highly variable anthropometric changes in later life may be detrimental to cognitive aging.

We further highlight that specific directions and patterns of anthropometric change are critical to understanding accelerated cognitive decline. Our study introduces the concept of cycling, defined as losing and then regaining in nutritional status in a specific period with respect to cognitive decline. Our study findings suggest that compared with the stable or gain groups, loss and cycling, particularly in BW, pose the greatest risk for cognitive health. Previous studies have reported results in the direction of nutritional status with cognitive function. For instance, a longitudinal study of 15,977 older adults without dementia found that changes in BMI—defined as the difference between the last and first BMI assessments—showed that both ≥5% increases and decreases, compared to stable BMI (<5% change), were associated with faster cognitive decline [17]. In another a longitudinal study of community‐dwelling older adults without dementia (aged >70 years old), researchers found that weight loss (≥5% decrease) during the first year of follow‐up predicted higher cognitive decline over a 5‐year follow‐up period [26]. A prior study using NHATS data, including 3 years of follow‐up, demonstrated that losses in BMI and WC were both related to an elevated risk of cognitive decline [13]. Our study is novel in examining the cycling of various anthropometric measures in relationship to cognitive decline. Analyzing the trajectory and pattern of longitudinal changes in BW and body composition offers deeper insights compared with focusing solely on the last‐to‐first assessment changes. These findings collectively suggest that both loss and cycling may be similarly detrimental to cognitive aging, emphasizing the importance of maintaining a stable nutritional status to mitigate cognitive decline in older adults.

Several potential mechanisms may help to explain the associations observed in our study. For instance, change in BMI, BW, or WC in older age might indicate underlying health issues such as frailty [27], functional decline [28], malnutrition [29], or chronic diseases (e.g., diabetes, cardiovascular outcomes) [30, 31], all of which can adversely impact cognitive health [32, 33, 34]. The cycle of losing and regaining weight could lead to metabolic stress [35] impacting cognition [36] through various mechanisms, such as insulin resistance [37] and oxidative stress [38]. Sarcopenia, a gradual loss of muscle mass and function, is a known risk factor of cognitive impairment [39, 40]. When adjusting for grip strength in the sensitivity analysis, a common but imperfect screening tool for sarcopenia, we observed slightly faster rates of cognitive decline across all pattern groups. This may be because individuals with relatively stable or gain patterns over time may also have low grip strength, potentially confounding the observed patterns. Another possible explanation is that low grip strength is independently associated with other unmeasured health conditions that contribute to cognitive decline. By accounting for grip strength, some of the shared variance through these alternative pathways may have been removed, thereby amplifying the observed relationship. A reverse relationship may be possible, as BW and body composition change may start several years before and after dementia is diagnosed [41]. In our sensitivity analysis, which excluded dementia cases that developed during follow‐up, the association was attenuated. However, the significant differences across variability quartiles and pattern groups remained largely consistent with those observed in the full sample, underscoring the robustness of our findings.

Intentional weight loss in older individuals with obesity or overweight may benefit cognitive function [42]. However, unintentional weight loss may be detrimental, as evidence has suggested that obesity in later life is associated with a lower risk of dementia [43], highlighting the potentially protective role of maintaining BW in older adults. We did not observe any differential associations between variability or patterns in nutritional status and cognitive trajectory based on the intentionality of weight loss. This may be because only about one‐half of the included participants ever reported the intentionality of their weight loss during the follow‐up period, resulting in limited statistical power. In our cohort, intentional weight loss may reflect lifestyle changes, whereas unintentional weight loss in older adults could indicate underlying illnesses or carry distinct cognitive implications [44]. Although we adjusted for the presence of several chronic conditions (e.g., cancer, diabetes, heart disease) and socioeconomic variables in our analysis, we were unable to fully account for the potential confounding effects of acute illnesses, medication side effects, other mental health conditions (e.g., depression, delirium, loneliness), or changes in the home environment (e.g., social problems or relationships) on BW fluctuations and cognitive decline [45]. Future studies should address these factors, potentially by linking to external databases (e.g., electronic medical records) or incorporating additional data collection during follow‐up.

This study has several strengths to consider. Our longitudinal investigation spanned up to 11 years and included multiple rounds of data, allowing a comprehensive understanding of nutritional status and cognitive changes. Objective measures were used for WC and cognitive function across three domains. The inclusion of BMI, BW, and WC offered insights into not only BW but also general obesity and central adiposity. Additionally, we used several metrics to compare and assess the magnitude, direction, and pattern of nutritional status variation. We also conducted several sensitivity analyses with robust results.

Our study has several limitations. First, as an observational study, we cannot draw causal inferences from our findings. Residual confounding may persist, as not all potential factors were captured by NHATS. For instance, detailed information on dietary habits (e.g., food intake) and comprehensive assessment of physical activity were lacking; participants were asked to list their favorite activities, but no data were collected on the intensity or duration of these activities. Additionally, the lack of direct measures of lean mass or body adiposity or diagnosis of sarcopenia further limits our capacity to explore the mechanisms underlying the variability and trajectory of anthropometric measures in relation to cognitive aging. Finally, the generalizability of our findings may be limited, as there are significant differences between the included and excluded participants in NHATS. It is crucial to consider other racial and ethnic groups in future research to ensure broader applicability of these associations.

## CONCLUSION

In conclusion, we observed accelerated rates of cognitive decline associated with greater variability and loss patterns in BW, BMI, and WC among older adults. A cycling pattern in BW in later life also indicates a heightened risk for faster cognitive decline. Our study findings emphasize the importance of maintaining stable nutritional status in older age and monitoring changes in anthropometric measures over time.

## AUTHOR CONTRIBUTIONS

Ashley C. Flores, Alexandra M. Wennberg, Cindy W. Leung, and Muzi Na designed the research; Ashley C. Flores and Muzi Na analyzed the data; Ashley C. Flores wrote the manuscript; Ashley C. Flores, Alexandra M. Wennberg, Cindy W. Leung, and Muzi Na reviewed and revised the manuscript; and Muzi Na had primary responsibility for the final content. All authors have read and approved the final manuscript.

## CONFLICT OF INTEREST STATEMENT

Alexandra M. Wennberg has received partial funding support from the external engagement office, Karolinska Institutet, on a collaborative project with Janssen Pharmaceutica NV and from the Gun & Bertil Stohne's Stifelse grant unrelated to the current manuscript. The other authors declared no conflicts of interest.

## Supporting information


**Figure S1.** Flowchart of study participant inclusion.
**Table S1.** The predicted trajectory of cognitive function z‐score between 2011 and 2021 in NHATS older adults by nutritional status variability quartiles excluding participants who developed dementia during the follow‐up period.
**Table S2.** The predicted trajectory of cognitive function z‐score between 2011 and 2021 in NHATS older adults by nutritional status variability quartiles excluding participants who developed diabetes during the follow‐up period.
**Table S3.** The predicted trajectory of cognitive function z‐score between 2011 and 2021 in NHATS older adults by nutritional status variability quartiles, adjusted for baseline grip strength additionally.
**Table S4.** The predicted trajectory of cognitive function z‐score between 2011 and 2021 in NHATS older adults by nutritional status last‐to‐first assessment change groups excluding participants who developed dementia during the follow‐up period.
**Table S5.** The predicted trajectory of cognitive function z‐score between 2011 and 2021 in NHATS older adults by nutritional status last‐to‐first assessment change groups excluding participants who developed diabetes during the follow‐up period.
**Table S6.** The predicted trajectory of cognitive function z‐score between 2011 and 2021 in NHATS older adults by nutritional status last‐to‐first assessment change groups, adjusted for baseline grip strength additionally.
**Table S7.** The predicted trajectory of cognitive function z‐score between 2011 and 2021 in NHATS older adults by nutritional status pattern excluding participants who developed dementia during the follow‐up period.
**Table S8.** The predicted trajectory of cognitive function z‐score between 2011 and 2021 in NHATS older adults by nutritional status pattern excluding participants who developed diabetes during the follow‐up period.
**Table S9.** The predicted trajectory of cognitive function z‐score between 2011 and 2021 in NHATS older adults by nutritional status pattern, adjusted for baseline grip strength additionally.
**Table S10.** Nutritional status variability metrics of BMI, BW, and WC by sex between 2011 and 2021 in NHATS older adults.
**Table S11.** Baseline characteristics of the included and excluded participants in NHATS.
**Table S1.** The predicted trajectory of cognitive function z‐score between 2011 and 2021 in NHATS older adults by nutritional status variability quartiles excluding participants who developed dementia during the follow‐up period.
**Table S2.** The predicted trajectory of cognitive function z‐score between 2011 and 2021 in NHATS older adults by nutritional status variability quartiles excluding participants who developed diabetes during the follow‐up period.
**Table S3.** The predicted trajectory of cognitive function z‐score between 2011 and 2021 in NHATS older adults by nutritional status variability quartiles, adjusted for baseline grip strength additionally.
**Table S4.** The predicted trajectory of cognitive function z‐score between 2011 and 2021 in NHATS older adults by nutritional status last‐to‐first assessment change groups excluding participants who developed dementia during the follow‐up period.
**Table S5.** The predicted trajectory of cognitive function z‐score between 2011 and 2021 in NHATS older adults by nutritional status last‐to‐first assessment change groups excluding participants who developed diabetes during the follow‐up period.
**Table S6.** The predicted trajectory of cognitive function z‐score between 2011 and 2021 in NHATS older adults by nutritional status last‐to‐first assessment change groups, adjusted for baseline grip strength additionally.
**Table S7.** The predicted trajectory of cognitive function z‐score between 2011 and 2021 in NHATS older adults by nutritional status pattern excluding participants who developed dementia during the follow‐up period.
**Table S8.** The predicted trajectory of cognitive function z‐score between 2011 and 2021 in NHATS older adults by nutritional status pattern excluding participants who developed diabetes during the follow‐up period.
**Table S9.** The predicted trajectory of cognitive function z‐score between 2011 and 2021 in NHATS older adults by nutritional status pattern, adjusted for baseline grip strength additionally.
**Table S10.** Nutritional status variability metrics of BMI, BW, and WC by sex between 2011 and 2021 in NHATS older adults.
**Table S11.** Baseline characteristics of the included and excluded participants in NHATS.

## Data Availability

The data referenced and described in the manuscript are publicly and freely available from the National Health and Aging Trends Study (https://www.nhats.org/researcher/data-access). The analytic code will be provided upon request, pending application and approval by the corresponding author, Dr. Muzi Na.
