# Supplementary material for: Variability in body weight and body composition and cognitive trajectories in older adults in the United States
Source: Obesity (Silver Spring). 2025 Jul 3;33(Suppl 1):122–30. doi: 10.1002/oby.24309 (PMC12592765; doi:10.1002/oby.24309)
Supplement: Supplementary file 1 — Figure S1. Flowchart of study participant inclusion. Table S1. The predicted trajectory of cognitive function z‐score between 2011 and 2021 in NHATS older adults by nutritional status variability quartiles excluding participants who developed dementia during the follow‐up period. Table S2. The predicted trajectory of cognitive function z‐score between 2011 and 2021 in NHATS older adults by nutritional status variability quartiles excluding participants who developed diabetes during the follow‐up period. Table S3. The predicted trajectory of cognitive function z‐score between 2011 and 2021 in NHATS older adults by nutritional status variability quartiles, adjusted for baseline grip strength additionally. Table S4. The predicted trajectory of cognitive function z‐score between 2011 and 2021 in NHATS older adults by nutritional status last‐to‐first assessment change groups excluding participants who developed dementia during the follow‐up period. Table S5. The predicted trajectory of cognitive function z‐score between 2011 and 2021 in NHATS older adults by nutritional status last‐to‐first assessment change groups excluding participants who developed diabetes during the follow‐up period. Table S6. The predicted trajectory of cognitive function z‐score between 2011 and 2021 in NHATS older adults by nutritional status last‐to‐first assessment change groups, adjusted for baseline grip strength additionally. Table S7. The predicted trajectory of cognitive function z‐score between 2011 and 2021 in NHATS older adults by nutritional status pattern excluding participants who developed dementia during the follow‐up period. Table S8. The predicted trajectory of cognitive function z‐score between 2011 and 2021 in NHATS older adults by nutritional status pattern excluding participants who developed diabetes during the follow‐up period. Table S9. The predicted trajectory of cognitive function z‐score between 2011 and 2021 in NHATS older adults by nutritional status pattern, adjusted [file OBY-33-122-s001.docx]

**Online Supporting Material**

**Figure S1.** Flow chart of study participant inclusion

**Table S1.** The predicted trajectory of cognitive function z-score between 2011 and 2021 in NHATS older adults by nutritional status variability quartiles excluding participants who developed dementia during the follow-up period

**Table S2.** The predicted trajectory of cognitive function z-score between 2011 and 2021 in NHATS older adults by nutritional status variability quartiles excluding participants who developed diabetes during the follow-up period

**Table S3.** The predicted trajectory of cognitive function z-score between 2011 and 2021 in NHATS older adults by nutritional status variability quartiles, adjusted for baseline grip strength additionally

**Table S4.** The predicted trajectory of cognitive function z-score between 2011 and 2021 in NHATS older adults by nutritional status last-to-first assessment change groups excluding participants who developed dementia during the follow-up period

**Table S5.** The predicted trajectory of cognitive function z-score between 2011 and 2021 in NHATS older adults by nutritional status last-to-first assessment change groups excluding participants who developed diabetes during the follow-up period

**Table S6.** The predicted trajectory of cognitive function z-score between 2011 and 2021 in NHATS older adults by nutritional status last-to-first assessment change groups, adjusted for baseline grip strength additionally

**Table S7.** The predicted trajectory of cognitive function z-score between 2011 and 2021 in NHATS older adults by nutritional status pattern excluding participants who developed dementia during the follow-up period

**Table S8.** The predicted trajectory of cognitive function z-score between 2011 and 2021 in NHATS older adults by nutritional status pattern excluding participants who developed diabetes during the follow-up period

**Table S9.** The predicted trajectory of cognitive function z-score between 2011 and 2021 in NHATS older adults by nutritional status pattern, adjusted for baseline grip strength additionally

**Table S10.** Nutritional status variability metrics of BMI, BW, and WC by sex between 2011 and 2021 in NHATS older adults

Table S11. Baseline characteristics of the included and excluded participants in NHATS


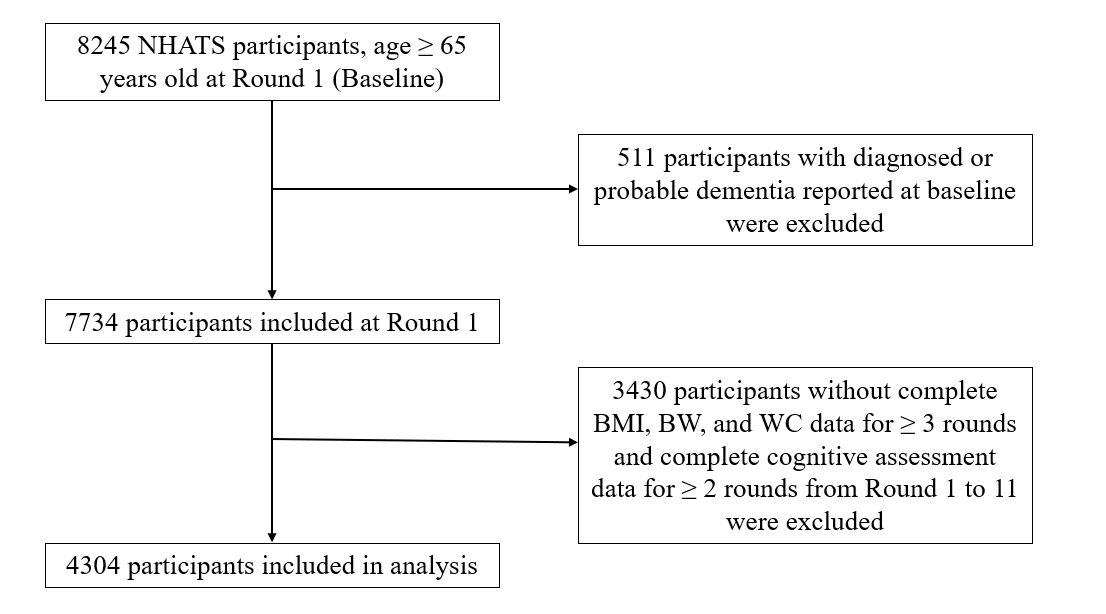


**Figure S1**. **Flow chart of study participant inclusion**

BMI, Body Mass Index; BW, Body Weight; NHATS, The National Health and Aging Trends Study; WC, Waist Circumference.

**Table S1. The predicted trajectory of cognitive function z-score between 2011 and 2021 in NHATS older adults by nutritional status variability quartiles excluding participants who developed dementia during the follow-up period**


|  |  | **Quartile 1** | **Quartile 2** | **Quartile 3** | **Quartile 4** | **P-interaction^4^** |
| --- | --- | --- | --- | --- | --- | --- |
|  | N | **β (95% CI)** | **β (95% CI)** | **β (95% CI)** | **β (95% CI)** |  |
| **BMI^1^** |  |  |  |  |  |  |
| SD | 4,184 | -0.007 (-0.013, -0.002) | -0.016 (-0.022, -0.011) | -0.020 (-0.026, -0.015) | -0.030 (-0.035, -0.024) | <0.0001 |
| CV | 4,184 | -0.005 (-0.010, 0.001) | -0.017 (-0.022, -0.012) | -0.019 (-0.025, -0.014) | -0.034 (-0.040, -0.028) | <0.0001 |
| RMSE | 4,189 | -0.010 (-0.015, -0.004) | -0.013 (-0.018, -0.007) | -0.021 (-0.026, -0.015) | -0.028 (-0.034, -0.022) | <0.0001 |
| **BW^1,2^** |  |  |  |  |  |  |
| SD | 4,188 | -0.010 (-0.015, -0.004) | -0.015 (-0.021, -0.010) | -0.021 (-0.026, -0.016) | -0.027 (-0.033, -0.021) | <0.0001 |
| CV | 4,188 | -0.009 (-0.015, -0.004) | -0.013 (-0.018, -0.007) | -0.021 (-0.027, -0.016) | -0.033 (-0.039, -0.027) | <0.0001 |
| RMSE | 4,192 | -0.009 (-0.015, -0.004) | -0.013 (-0.018, -0.008) | -0.022 (-0.028, -0.017) | -0.026 (-0.032, -0.021) | <0.0001 |
| **WC^1,3^** |  |  |  |  |  |  |
| SD | 3,915 | -0.008 (-0.014, -0.003) | -0.016 (-0.021, -0.010) | -0.016 (-0.022, -0.011) | -0.022 (-0.028, -0.016) | 0.0007 |
| CV | 3,915 | -0.009 (-0.015, -0.003) | -0.014 (-0.019, -0.008) | -0.015 (-0.021, -0.010) | -0.025 (-0.031, -0.019) | <0.0001 |
| RMSE | 3,919 | -0.008 (-0.014, -0.002) | -0.017 (-0.023, -0.012) | -0.018 (-0.023, -0.012) | -0.017 (-0.022, -0.011) | 0.0163 |

BMI, Body Mass Index; BW, Body Weight; CI, Confidence Intervals; CV, Coefficient of Variation; NHATS, The National Health and Aging Trends Study; RMSE, Root Mean Square Error; SD, Standard Deviation; WC, Waist Circumference.

^1^ Adjusted for baseline covariates including age, sex, race/ethnicity, education levels, regular smoker status, nutritional status being examined, number of follow-ups, and time-varying covariates for marital status, imputed income quintiles, current smoking status, depression score, anxiety score, current diagnosis status of heart disease, diabetes, hypertension, stroke, or cancer.

^2^ Further adjusted for baseline height.

^3^ Further adjusted for baseline BMI.

^4^ P-interaction indicates the p-value of the interaction between quartile group and time and tested at a significance level of 0.05.

**Table S2. The predicted trajectory of cognitive function z-score between 2011 and 2021 in NHATS older adults by nutritional status variability quartiles excluding participants who developed diabetes during the follow-up period**

|  |  | **Quartile 1** | **Quartile 2** | **Quartile 3** | **Quartile 4** | **P-interaction^4^** |
| --- | --- | --- | --- | --- | --- | --- |
|  | N | **β (95% CI)** | **β (95% CI)** | **β (95% CI)** | **β (95% CI)** |  |
| **BMI^1^** |  |  |  |  |  |  |
| SD | 3,166 | -0.010 (-0.016, -0.003) | -0.019 (-0.025, -0.012) | -0.025 (-0.032, -0.019) | -0.039 (-0.046, -0.032) | <0.0001 |
| CV | 3,166 | -0.007 (-0.013, -0.001) | -0.019 (-0.025, -0.013) | -0.025 (-0.032, -0.019) | -0.043 (-0.049, -0.036) | <0.0001 |
| RMSE | 3,169 | -0.012 (-0.019, -0.006) | -0.014 (-0.020, -0.008) | -0.026 (-0.032, -0.019) | -0.037 (-0.044, -0.030) | <0.0001 |
| **BW^1,2^** |  |  |  |  |  |  |
| SD | 3,170 | -0.013 (-0.019, -0.007) | -0.018 (-0.025, -0.012) | -0.026 (-0.033, -0.020) | -0.035 (-0.042, -0.028) | <0.0001 |
| CV | 3,170 | -0.012 (-0.018, -0.005) | -0.016 (-0.022, -0.009) | -0.028 (-0.034, -0.021) | -0.041 (-0.048, -0.034) | <0.0001 |
| RMSE | 3,172 | -0.011 (-0.018, -0.005) | -0.017 (-0.023, -0.010) | -0.029 (-0.035, -0.022) | -0.034 (-0.041, -0.027) | <0.0001 |
| **WC^1,3^** |  |  |  |  |  |  |
| SD | 2,971 | -0.014 (-0.021, -0.008) | -0.017 (-0.024, -0.011) | -0.019 (-0.026, -0.013) | -0.029 (-0.036, -0.022) | 0.0019 |
| CV | 2,971 | -0.015 (-0.022, -0.008) | -0.015 (-0.021, -0.008) | -0.018 (-0.024, -0.011) | -0.031 (-0.038, -0.024) | 0.0001 |
| RMSE | 2,974 | -0.015 (-0.022, -0.007) | -0.020 (-0.026, -0.014) | -0.019 (-0.025, -0.012) | -0.023 (-0.030, -0.016) | 0.2078 |


BMI, Body Mass Index; BW, Body Weight; CI, Confidence Intervals; CV, Coefficient of Variation; NHATS, The National Health and Aging Trends Study; RMSE, Root Mean Square Error; SD, Standard Deviation; WC, Waist Circumference.

^1^ Adjusted for baseline covariates including age, sex, race/ethnicity, education levels, regular smoker status, nutritional status being examined, number of follow-ups, and time-varying covariates for marital status, imputed income quintiles, current smoking status, depression score, anxiety score, current diagnosis status of heart disease, diabetes, hypertension, stroke, or cancer.

^2^ Further adjusted for baseline height.

^3^ Further adjusted for baseline BMI.

^4^ P-interaction indicates the p-value of the interaction between quartile group and time and tested at a significance level of 0.05.

**Table S3. The predicted trajectory of cognitive function z-score between 2011 and 2021 in NHATS older adults by nutritional status variability quartiles, , adjusted for baseline grip strength additionally**

|  |  | **Quartile 1** | **Quartile 2** | **Quartile 3** | **Quartile 4** | **P-interaction^4^** |
| --- | --- | --- | --- | --- | --- | --- |
|  | N | **β (95% CI)** | **β (95% CI)** | **β (95% CI)** | **β (95% CI)** |  |
| **BMI^1^** |  |  |  |  |  |  |
| SD | 4,093 | -0.014 (-0.020, -0.008) | -0.021 (-0.026, -0.015) | -0.029 (-0.034, -0.023) | -0.039 (-0.045, -0.033) | <0.0001 |
| CV | 4,093 | -0.011 (-0.017, -0.006) | -0.022 (-0.027, -0.016) | -0.027 (-0.032, -0.021) | -0.046 (-0.052, -0.040) | <0.0001 |
| RMSE | 4,099 | -0.016 (-0.022, -0.011) | -0.019 (-0.024, -0.013) | -0.028 (-0.033, -0.022) | -0.039 (-0.045, -0.033) | <0.0001 |
| **BW^1,2^** |  |  |  |  |  |  |
| SD | 4,097 | -0.016 (-0.022, -0.011) | -0.021 (-0.026, -0.015) | -0.029 (-0.034, -0.023) | -0.036 (-0.042, -0.030) | <0.0001 |
| CV | 4,097 | -0.015 (-0.020, -0.009) | -0.019 (-0.025, -0.014) | -0.028 (-0.033, -0.022) | -0.043 (-0.049, -0.038) | <0.0001 |
| RMSE | 4,101 | -0.015 (-0.021, -0.010) | -0.019 (-0.025, -0.014) | -0.030 (-0.036, -0.025) | -0.035 (-0.041, -0.030) | <0.0001 |
| **WC^1,3^** |  |  |  |  |  |  |
| SD | 3,909 | -0.015 (-0.020, -0.009) | -0.022 (-0.027, -0.016) | -0.024 (-0.029, -0.018) | -0.031 (-0.037, -0.025) | <0.0001 |
| CV | 3,909 | -0.015 (-0.021, -0.009) | -0.020 (-0.025, -0.014) | -0.022 (-0.027, -0.016) | -0.034 (-0.040, -0.028) | <0.0001 |
| RMSE | 3,913 | -0.016 (-0.022, -0.010) | -0.023 (-0.029, -0.017) | -0.025 (-0.030, -0.019) | -0.024 (-0.030, -0.018) | 0.0404 |

BMI, Body Mass Index; BW, Body Weight; CI, Confidence Intervals; CV, Coefficient of Variation; NHATS, The National Health and Aging Trends Study; RMSE, Root Mean Square Error; SD, Standard Deviation; WC, Waist Circumference.

^1^ Adjusted for baseline covariates including age, sex, race/ethnicity, education levels, regular smoker status, nutritional status being examined, number of follow-ups, and time-varying covariates for marital status, imputed income quintiles, current smoking status, depression score, anxiety score, current diagnosis status of heart disease, diabetes, hypertension, stroke, or cancer, and baseline grip strength.

^2^ Further adjusted for baseline height.

^3^ Further adjusted for baseline BMI.

^4^ P-interaction indicates the p-value of the interaction between quartile group and time and tested at a significance level of 0.05.


**Table S4. The predicted trajectory of cognitive function z-score between 2011 and 2021 in NHATS older adults by nutritional status last-to-first assessment change groups excluding participants who developed dementia during the follow-up period**

|  |  | **Loss** | **Stable** | **Gain** | **P-interaction^4^** |
| --- | --- | --- | --- | --- | --- |
|  | N | **β (95% CI)** | **β (95% CI)** | **β (95% CI)** |  |
| **BMI^1^** | 4,196 | -0.025 (-0.030, -0.020) | -0.011 (-0.016, -0.006) | -0.012 (-0.018, -0.006) | <0.0001 |
| **BW^1,2^** | 4,099 | -0.023 (-0.027, -0.018) | -0.011 (-0.015, -0.006) | -0.012 (-0.020, -0.005) | <0.0001 |
| **WC^1,3^** | 3,869 | -0.022 (-0.028, -0.017) | -0.013 (-0.018, -0.008) | -0.013 (-0.019, -0.008) | 0.0027 |

BMI, Body Mass Index; BW, Body Weight; CI, Confidence Intervals; NHATS, The National Health and Aging Trends Study; WC, Waist Circumference.

^1^ Adjusted for baseline covariates including age, sex, race/ethnicity, education levels, regular smoker status, nutritional status being examined, number of follow-ups, and time-varying covariates for marital status, imputed income quintiles, current smoker status, depression score, anxiety score, current diagnosis status of heart disease, diabetes, hypertension, stroke, or cancer.

^2^ Further adjusted for baseline height.

^3^ Further adjusted for baseline BMI.

^4^ P-interaction indicates the p-value of the interaction between last-to-first assessment change group and time and tested at a significance level of 0.05.


**Table S5. The predicted trajectory of cognitive function z-score between 2011 and 2021 in NHATS older adults by nutritional status last-to-first assessment change groups excluding participants who developed diabetes during the follow-up period**

|  |  | **Loss** | **Stable** | **Gain** | **P-interaction^4^** |
| --- | --- | --- | --- | --- | --- |
|  | N | **β (95% CI)** | **β (95% CI)** | **β (95% CI)** |  |
| **BMI^1^** | 3,175 | -0.029 (-0.034, -0.023) | -0.014 (-0.020, -0.009) | -0.017 (-0.025, -0.010) | <0.0001 |
| **BW^1,2^** | 3,098 | -0.026 (-0.031, -0.020) | -0.015 (-0.021, -0.010) | -0.019 (-0.028, -0.010) | 0.0009 |
| **WC^1,3^** | 2,937 | -0.026 (-0.032, -0.019) | -0.017 (-0.023, -0.012) | -0.018 (-0.024, -0.011) | 0.038 |

BMI, Body Mass Index; BW, Body Weight; CI, Confidence Intervals; NHATS, The National Health and Aging Trends Study; WC, Waist Circumference.

^1^ Adjusted for baseline covariates including age, sex, race/ethnicity, education levels, regular smoker status, nutritional status being examined, number of follow-ups, and time-varying covariates for marital status, imputed income quintiles, current smoker status, depression score, anxiety score, current diagnosis status of heart disease, diabetes, hypertension, stroke, or cancer.

^2^ Further adjusted for baseline height.

^3^ Further adjusted for baseline BMI.

^4^ P-interaction indicates the p-value of the interaction between last-to-first assessment change group and time and tested at a significance level of 0.05.


**Table S6. The predicted trajectory of cognitive function z-score between 2011 and 2021 in NHATS older adults by nutritional status last-to-first assessment change groups, adjusted for baseline grip strength additionally**

|  |  | **Loss** | **Stable** | **Gain** | **P-interaction^4^** |
| --- | --- | --- | --- | --- | --- |
|  | N | **β (95% CI)** | **β (95% CI)** | **β (95% CI)** |  |
| **BMI^1^** | 4,107 | -0.034 (-0.038, -0.029) | -0.018 (-0.023, -0.013) | -0.021 (-0.028, -0.015) | <0.0001 |
| **BW^1,2^** | 4,009 | -0.029 (-0.034, -0.024) | -0.018 (-0.023, -0.013) | -0.021 (-0.028, -0.013) | <0.0001 |
| **WC^1,3^** | 3,864 | -0.028 (-0.034, -0.023) | -0.019 (-0.024, -0.014) | -0.023 (-0.028, -0.017) | 0.0086 |

BMI, Body Mass Index; BW, Body Weight; CI, Confidence Intervals; NHATS, The National Health and Aging Trends Study; WC, Waist Circumference.

^1^ Adjusted for baseline covariates including age, sex, race/ethnicity, education levels, regular smoker status, nutritional status being examined, number of follow-ups, and time-varying covariates for marital status, imputed income quintiles, current smoker status, depression score, anxiety score, current diagnosis status of heart disease, diabetes, hypertension, stroke, or cancer, and baseline grip strength.

^2^ Further adjusted for baseline height.

^3^ Further adjusted for baseline BMI.

^4^ P-interaction indicates the p-value of the interaction between last-to-first assessment change group and time and tested at a significance level of 0.05.


**Table S7. The predicted trajectory of cognitive function z-score between 2011 and 2021 in NHATS older adults by nutritional status pattern excluding participants who developed dementia during the follow-up period**

|  |  | **Stable** | **Loss** | **Gain** | **Cycling** | **P-interaction^4^** |
| --- | --- | --- | --- | --- | --- | --- |
|  | N | **β (95% CI)** | **β (95% CI)** | **β (95% CI)** | **β (95% CI)** |  |
| **BMI^1^** | 4,173 | -0.012 (-0.021, -0.003) | -0.018 (-0.024, -0.012) | -0.012 (-0.018, -0.005) | -0.018 (-0.023, -0.014) | 0.1522 |
| **BW^1,2^** | 4,182 | -0.011 (-0.018, -0.003) | -0.018 (-0.023, -0.013) | -0.011 (-0.018, -0.004) | -0.019 (-0.024, -0.014) | 0.0269 |
| **WC^1,3^** | 3,901 | -0.005 (-0.015, 0.004) | -0.018 (-0.026, -0.010) | -0.013 (-0.019, -0.007) | -0.017 (-0.021, -0.012) | 0.0771 |

BMI, Body Mass Index; BW, Body Weight; CI, Confidence Intervals; NHATS, The National Health and Aging Trends Study; WC, Waist Circumference.

^1^ Adjusted for baseline covariates including age, sex, race/ethnicity, education levels, regular smoker status, nutritional status being examined, number of follow-ups, and time-varying covariates for marital status, imputed income quintiles, current smoker status, depression score, anxiety score, current diagnosis status of heart disease, diabetes, hypertension, stroke, or cancer.

^2^ Further adjusted for baseline height.

^3^ Further adjusted for baseline BMI.

^4^ P-interaction indicates the p-value of the interaction between nutritional status pattern group and time and tested at a significance level of 0.05.


**Table S8. The predicted trajectory of cognitive function z-score between 2011 and 2021 in NHATS older adults by nutritional status pattern excluding participants who developed diabetes during the follow-up period**

|  |  | **Stable** | **Loss** | **Gain** | **Cycling** | **P-interaction^4^** |
| --- | --- | --- | --- | --- | --- | --- |
|  | N | **β (95% CI)** | **β (95% CI)** | **β (95% CI)** | **β (95% CI)** |  |
| **BMI^1^** | 3,156 | -0.015 (-0.025, -0.004) | -0.021 (-0.028, -0.014) | -0.017 (-0.024, -0.009) | -0.022 (-0.028, -0.017) | 0.2600 |
| **BW^1,2^** | 3,165 | -0.014 (-0.023, -0.006) | -0.021 (-0.027, -0.015) | -0.019 (-0.027, -0.010) | -0.024 (-0.029, -0.018) | 0.1470 |
| **WC^1,3^** | 2,960 | -0.008 (-0.020, 0.004) | -0.021 (-0.031, -0.012) | -0.018 (-0.025, -0.011) | -0.020 (-0.026, -0.015) | 0.1897 |

BMI, Body Mass Index; BW, Body Weight; CI, Confidence Intervals; NHATS, The National Health and Aging Trends Study; WC, Waist Circumference.

^1^ Adjusted for baseline covariates including age, sex, race/ethnicity, education levels, regular smoker status, nutritional status being examined, number of follow-ups, and time-varying covariates for marital status, imputed income quintiles, current smoker status, depression score, anxiety score, current diagnosis status of heart disease, diabetes, hypertension, stroke, or cancer.

^2^ Further adjusted for baseline height.

^3^ Further adjusted for baseline BMI.

^4^ P-interaction indicates the p-value of the interaction between nutritional status pattern group and time and tested at a significance level of 0.05.

**Table S9. The predicted trajectory of cognitive function z-score between 2011 and 2021 in NHATS older adults by nutritional status pattern, adjusted for baseline grip strength additionally**

|  |  | **Stable** | **Loss** | **Gain** | **Cycling** | **P-interaction^4^** |
| --- | --- | --- | --- | --- | --- | --- |
|  | N | **β (95% CI)** | **β (95% CI)** | **β (95% CI)** | **β (95% CI)** |  |
| **BMI^1^** | 4,082 | -0.020 (-0.029, -0.010) | -0.026 (-0.032, -0.020) | -0.019 (-0.026, -0.012) | -0.025 (-0.030, -0.021) | 0.1604 |
| **BW^1,2^** | 4,091 | -0.017 (-0.025, -0.009) | -0.024 (-0.029, -0.019) | -0.019 (-0.027, -0.012) | -0.027 (-0.032, -0.022) | 0.0348 |
| **WC^1,3^** | 3,895 | -0.012 (-0.022, -0.002) | -0.025 (-0.033, -0.017) | -0.021 (-0.028, -0.015) | -0.023 (-0.028, -0.019) | 0.1207 |

BMI, Body Mass Index; BW, Body Weight; CI, Confidence Intervals; NHATS, The National Health and Aging Trends Study; WC, Waist Circumference.

^1^ Adjusted for baseline covariates including age, sex, race/ethnicity, education levels, regular smoker status, nutritional status being examined, number of follow-ups, and time-varying covariates for marital status, imputed income quintiles, current smoker status, depression score, anxiety score, current diagnosis status of heart disease, diabetes, hypertension, stroke, or cancer, and baseline grip strength.

^2^ Further adjusted for baseline height.

^3^ Further adjusted for baseline BMI.

^4^ P-interaction indicates the p-value of the interaction between nutritional status pattern group and time and tested at a significance level of 0.05.


**Table S10. Nutritional status variability metrics of BMI, BW, and WC by sex between 2011 and 2021 in NHATS older adults**

|  | N | **P-interaction by sex^4^** | N | **P-interaction by intentional weight loss^5^** |
| --- | --- | --- | --- | --- |
| **BMI^1^** |  |  |  |  |
| SD | 4,191 | 0.4502 | 2,085 | 0.3076 |
| CV | 4,191 | 0.2063 | 2,085 | 0.3145 |
| RMSE | 4,196 | 0.1374 | 2,085 | 0.3557 |
| First to Last | 4,203 | 0.3928 | 2,088 | 0.2313 |
| Pattern | 4,180 | 0.6720 | 2,080 | **0.0398** |
| **BW^1,2^** |  |  |  |  |
| SD | 4,195 | 0.2237 | 2,086 | 0.8033 |
| CV | 4,195 | **0.0340** | 2,086 | 0.4254 |
| RMSE | 4,199 | 0.0511 | 2,086 | 0.1626 |
| First to Last | 4,105 | 0.3681 | 2,048 | 0.3303 |
| Pattern | 4,189 | 0.4018 | 2,084 | 0.3618 |
| **WC^1,3^** |  |  |  |  |
| SD | 3,920 | **0.0115** | 1,963 | 0.8313 |
| CV | 3,920 | 0.0794 | 1,963 | 0.7122 |
| RMSE | 3,925 | 0.1090 | 1,965 | 0.4973 |
| First to Last | 3,876 | 0.4090 | 1,947 | 0.2756 |
| Pattern | 3,906 | 0.6775 | 1,954 | 0.8313 |

BMI, Body Mass Index; BW, Body Weight; CI, Confidence Intervals; NHATS, The National Health and Aging Trends Study; WC, Waist Circumference.

^1^ Adjusted for baseline covariates including age, sex, race/ethnicity, education levels, regular smoker status, nutritional status being examined, number of follow-ups, and time-varying covariates for marital status, imputed income quintiles, current smoker status, depression score, anxiety score, current diagnosis status of heart disease, diabetes, hypertension, stroke, or cancer.

^2^ Further adjusted for baseline height.

^3^ Further adjusted for baseline BMI.

^4^ P-interaction indicates the p-value of the interaction between each exposure, wave, and sex and tested at a significance level of 0.05.

^5^ P-interaction indicates the p-value of the interaction between each exposure, wave, and intentional weight loss (yes or no) and tested at a significance level of 0.05.

Table S11. Baseline characteristics of the included and excluded participants in NHATS

|  | **Included** | | **Excluded** | | **P-value^4^** |
| --- | --- | --- | --- | --- | --- |
|  | **N** | **% or Mean (SE)** | **N** | **% or Mean (SE)** |  |
| Age groups, % | 4,304 |  | 3,941 |  | <0.0001 |
| 65-69 y |  | 30.6 |  | 22.6 |  |
| 70-74 y |  | 26.3 |  | 22.2 |  |
| 75-79 y |  | 19.2 |  | 18.5 |  |
| 80-84 y |  | 14 |  | 15.9 |  |
| 85-89 y |  | 7.3 |  | 12.7 |  |
| ≥ 90 y |  | 2.7 |  | 8.0 |  |
| Sex, % | 4,304 |  | 3,941 |  | 0.4497 |
| Male |  | 43.3 |  | 42.4 |  |
| Female |  | 56.7 |  | 57.7 |  |
| Race/Ethnicity, % | 4,296 |  | 3,862 |  | <0.0001 |
| White, non-Hispanic |  | 84.5 |  | 77.0 |  |
| Black, non-Hispanic |  | 7.4 |  | 9.6 |  |
| Hispanic |  | 5.5 |  | 4.9 |  |
| Other |  | 2.6 |  | 8.5 |  |
| Education, % | 4,298 |  | 3,215 |  | <0.0001 |
| < High school diploma |  | 17.6 |  | 28.1 |  |
| High school diploma |  | 26.5 |  | 29.3 |  |
| > High school diploma |  | 55.9 |  | 42.6 |  |
| Smoking Status, % | 4,304 |  | 3,292 |  | 0.1008 |
| Never smoker |  | 47.8 |  | 46.7 |  |
| Ever smoker |  | 44.5 |  | 43.7 |  |
| Current smoker |  | 7.7 |  | 9.6 |  |
| Marital Status, % | 4,300 |  | 3,300 |  | 0.0006 |
| Married, living with partner |  | 59.2 |  | 53.8 |  |
| Not married, widowed, separated, divorced |  | 40.8 |  | 46.2 |  |
| Imputed Income Quintiles, % | 4,304 |  | 3,305 |  | <0.0001 |
| 1 ($0-$9,000) |  | 17.4 |  | 25.9 |  |
| 2 ($9,001-$17,402) |  | 16.6 |  | 20.3 |  |
| 3 ($17,403-$30,000) |  | 19.2 |  | 20.1 |  |
| 4 ($30,001-$55,000) |  | 22.4 |  | 19.4 |  |
| 5 (>$55,000) |  | 24.4 |  | 14.4 |  |
| Depression, score^2^ | 4,285 | 2.8 (0.02) | 3,425 | 3.2 (0.04) | <0.0001 |
| Anxiety, score^3^ | 4,295 | 2.8 (0.02) | 3,434 | 3.1 (0.03) | <0.0001 |
| Heart disease, % | 4,298 | 16.2 | 3,295 | 19.3 | 0.0036 |
| Diabetes, % | 4,304 | 22.8 | 3,302 | 25.4 | 0.0281 |
| Hypertension, % | 4,298 | 63.4 | 3,301 | 64.8 | 0.3201 |
| Stroke, % | 4,301 | 8.4 | 3,300 | 12.4 | <0.0001 |
| Cancer, % | 4,302 | 25.5 | 3,304 | 26.3 | 0.5519 |
| BMI, kg/m^2^ | 4,247 | 27.9 (0.11) | 3,253 | 27.1 (0.15) | <0.0001 |
| BW, lb | 4,274 | 174.0 (0.84) | 3,323 | 166.9 (0.97) | <0.0001 |
| WC, in | 4,140 | 39.4 (0.22) | 2,785 | 39.4 (0.20) | 0.903 |

BMI, Body Mass Index; NHATS, The National Health and Aging Trends Study

^1^ Adjusted for the complex sampling scheme

^2^ Depressive symptoms score was calculated based on the validated 2-item Patient Health Questionnaire

^3^ Anxiety score was calculated based on the validated 2-item Generalized Anxiety Disorder Scale

^4^ P-values are generated from the Pearson's chi-squared test.
